# A systematic review and meta-analysis on anthelmintic control programs for *Echinococcus multilocularis* in wild and domestic carnivores

**DOI:** 10.1016/j.fawpar.2019.e00042

**Published:** 2019-03-14

**Authors:** Gérald Umhang, Alessia Possenti, Vittoria Colamesta, Silvia d'Aguanno, Giuseppe La Torre, Franck Boué, Adriano Casulli

**Affiliations:** a ANSES, Wildlife Surveillance and Eco-epidemiology unit, Technopôle Agricole et Vétérinaire, B.P. 40009, 54220 Malzéville, France; b European Union Reference Laboratory for Parasites (EURLP), Department of Infectious Diseases, Istituto Superiore di Sanità (ISS), Rome, Italy; c Sapienza University of Rome, Department of Public Health and Infectious Diseases, Rome, Italy; d World Health Organization Collaborating Centre for the Epidemiology, Detection and Control of Cystic and Alveolar Echinococcosis (in humans and animals), Department of Infectious Diseases, Istituto Superiore di Sanità (ISS), Rome, Italy

**Keywords:** *Echinococcus multilocularis*, systematic review, control programs, baiting

## Abstract

Human alveolar echinococcosis (AE), caused by the tapeworm *Echinococcus multilocularis,* is one of the most dangerous zoonoses in the Northern hemisphere. In Europe, the parasite's life cycle is sylvatic, involving small rodents as intermediate hosts and red foxes as the major definitive hosts. Given the severity of this disease in humans and the high levels of environmental contamination with *E. multilocularis* in endemic areas, it seems crucial to implement control measures in order to prevent human AE. This systematic review identifies previous anthelmintic control programs targeting *E. multilocularis* in wild and domestic carnivores and evaluates the effectiveness of the different strategies implemented. A search through six databases identified 302 scientific papers for the period 1950–2015, of which only 17 were retained according to the inclusion criteria set. These 17 papers focused on control of *E. multilocularis* by baiting foxes in highly endemic areas of Europe or Japan, with the exception of one study focused on dogs in Alaska. The papers highlighted differences in baiting types, baiting frequency, choice of control areas and length of treatment period. Overall, these studies resulted in a sharp and statistically significant decrease in parasite prevalence, confirmed by the absence of overlap between confidence intervals for the pooled risk differences of control and treated areas. A monthly baiting frequency was proven to be highly effective at decreasing *E. multilocularis* prevalence in foxes, even in highly endemic areas and in a short period of time. Nevertheless, when foxes were not fully dewormed, the parasite showed a strong capacity to rapidly recover its initial prevalence.

The fox baiting approach appears to be the most useful method for controlling the sylvatic life cycle of *E. multilocularis*, but it require a cost/benefit analysis before it is likely to be accepted by stakeholders.

## Highlights

• This systematic review evaluated the effectiveness of the control programmes of *E. multilocularis.*
• Differences in baiting types, baiting frequency, selection of control areas and treatment period were highlighted.
• A monthly baiting was proved to be efficient to decrease *E. multilocularis* prevalence in foxes.

## 1 Introduction

Human alveolar echinococcosis (AE), caused by the tapeworm *Echinococcus multilocularis,* is a life-threatening parasitic zoonosis found in the Northern hemisphere (Budke et al., 2017). In humans, the disease is characterized by the slow growth of a primary tumor-like parasitic lesion, usually located in the liver (Brunetti et al., 2010). The disease's long asymptomatic period of around 5–15 years complicates the process of understanding its transmission pathways and associated risk factors (Possenti et al., 2016). In 2010, it was estimated that there were approximately 18,235 (CIs 11,900–28,200) new cases of human AE per annum worldwide, of which 16,629 (91%) occurred in China and 1606 outside China (Torgerson et al., 2010). A global estimation of the annual loss caused by AE was evaluated at 650,000 disability-adjusted life years (Torgerson and Craig, 2011). Nowadays, these figures should be considered an underestimate, particularly in light of new high-endemic foci being detected, such as those in Kyrgyzstan (Bebezov et al., 2018) and in China's Western Gansu Province (Han et al., 2015). Humans are aberrant hosts and become infected by orally ingesting *E. multilocularis* eggs from the environment. The parasite's life cycle involves small rodents that harbor the larval stage of the parasite in their liver. These larvae are then ingested by the definitive hosts—canids—through a predator-prey system. Once the metacestodes are ingested, they develop into adult worms in the small intestines of canids and eggs are then excreted in definitive host feces. Red foxes (*Vulpes vulpes*) are the main definitive hosts in Europe, but domestic dogs and other wild canids such as raccoon dogs (*Nyctereutes procyonoides*), arctic foxes (*Vulpes lagopus*), golden jackals (*Canis aureus*), eurasian wolves (*Canis lupus lupus*) and coyotes (*Canis latrans*) can also be infected (Oksanen et al., 2016).

Over the past two decades, intensive epidemiological research has suggested significant geographical spread of the parasite from Central to Northern, Eastern, and Western Europe (Gottstein et al., 2015). Simultaneously, an increase in *E. multilocularis* prevalence in red foxes has been reported in historically endemic areas of Europe (Berke et al., 2008; Combes et al., 2012; König et al., 2005). An increase in red fox populations along with socio-economic and ecological changes are likely to have fostered this spread (Hegglin and Deplazes, 2013). These factors have also increased the parasite's prevalence in urban areas due to foxes colonizing European and Japanese cities (Deplazes et al., 2004; Robardet et al., 2008; Tsukada et al., 2000). In Europe, the level of environmental contamination with *E. multilocularis* eggs has been estimated to be highest in peri-urban areas, where rural and urban habitats intersect (Deplazes et al., 2004). The intensive use of these areas by humans for recreational purposes constitutes a major threat to human health. Nevertheless, the multitude of urban and peri-urban landscapes in endemic areas of the world entails a variety of circumstances affecting parasite circulation and infection pressure; these factors do not necessarily follow a linear gradient from city center to surroundings (Liccioli et al., 2015).

Given AE's severity and the high level of environmental contamination with *E. multilocularis* eggs in endemic areas, control measures should be implemented to eliminate or at least prevent this parasitic infection from continuing to spread. No vaccines against *E. multilocularis* will be available for humans or animals in the foreseeable future (Takahashi et al., 2013). In addition, fox culling is questionable in terms of ethical concerns and effectiveness (Comte et al., 2017). For the above-mentioned reasons, controlling the parasite by baiting foxes currently appears to be the most effective way to limit environmental contamination with *E. multilocularis* eggs. Successful anthelmintic campaigns using praziquantel have previously targeted dogs in order to control *E. granulosus* in many parts of the world (Schelling et al., 1997). The possibility of incorporating this anthelmintic drug in attractive baits has enabled *E. multilocularis* control programs to be designed specifically for foxes.

This systematic review (SR) aims to identify past anthelmintic control programs for *E. multilocularis* in wild and domestic carnivores and to evaluate the effectiveness of the different strategies implemented. By comparing anthelmintic protocols and their associated effects on *E. multilocularis* prevalence, we may identify key success factors and the feasibility of implementing such control programs.

## 2 Materials and methods

### 2.1 Search strategy and data extraction

This SR was conducted according to PRISMA guidelines (Moher et al., 2009). The databases screened in the literature search were MEDLINE (PubMed), EMBASE (Excerpta Medica Database), SCI SEARCH (Science Citation Index), BIOSIS (Biological Abstracts), CABI (Centre for Agricultural Bioscience International), and GOOGLE SCHOLAR. The platform used for the search was STN International (Fachinformationszentrum Karlsruhe—FIZ Karlsruhe—available online at http://www.fiz-karlsruhe.de/stn.html?&L=1). When keywords were entered in the search process, a hashtag (#) and/or question mark (?) were included to expand the search in order to include words with the same root but different spellings, varying by one letter (#) or more (?). These search terms were combined with the Boolean operators (and/or). The full electronic search strategy, including any limits used, was: [echinococcus multilocularis or (echinocococcus and multilocularis) or e# multilocularis or alveolar echinococcosis or a# echinococcosis] and (dog# or fox or foxes or canis or canid? or vulpes or domestic or sylvatic? or wild?) and (anthelmintic# or antihelmintic# or praziquantel or deworm? or de worm? or antinematod? or vermifuge# or bait? or anti helmintic# or antihelmint?) and (program? or eradicat? or control? or eliminat?). Only papers published between 1950 and 11/02/2015 were included in this SR. The papers were written in English, German, French, Polish, Finnish, Dutch, Spanish, or Italian. Studies were excluded if they lacked original data (e.g. reviews) or involved the wrong agent (i.e. *Echinococcus granulosus*) or host (i.e. humans). Duplicates between databases were removed and the study selection process was carried out by two independent researchers from start to finish; any disagreements were resolved by consensus between the two researchers. An initial screening was undertaken according to the title and abstract's relevance in terms of the focus of this study. The full texts of the shortlisted papers were examined through a second screening stage in order to assess their eligibility. For each retained paper, data on the following were extracted into tables: the article reference, the period and geographic location of the trial, the eradication method, the diagnostic method applied to animal samples, and the associated results. If more than one area was analyzed in a single study, each area was considered a single sub-study, irrespective of the protocol used. The studies' quality was assessed using the Cochrane Collaboration's tool for assessing the risk of bias (Higgins et al., 2011). The potential presence of bias from six domains was evaluated under the following categories: sequence generation, allocation concealment, blinding, incomplete outcome data, selective outcome reporting, and other sources of bias.

### 2.2 Meta-analysis

Meta-analysis was performed across studies if at least two similar studies were available. Studies were included only if data were available on the sample size and number of positive samples both before and after the treatment. In line with this approach, the pooled risk difference (RD) was calculated for both baiting and control areas by analyzing the difference between the event rate (*E. multilocularis*-positive) at the start and end of the study. The 95% confidence intervals (95% CI) of RD obtained from baiting and control areas were subsequently compared. If there was overlap between the 95% CI of the two areas, the difference was not considered statistically significant. Meta-analysis was conducted using statistical software Stats Direct 2.8.0 (StatsDirect Ltd., Altrincham, UK). A Cochran's Q test was carried out to assess the degree of heterogeneity between studies, and the *I*^2^ statistic was used to describe the percentage of total variation across the studies as a result of heterogeneity. If the *p*-value of this Q test was <0.05 and *I*^2^ was >50%, heterogeneity was assumed and a random-effect model used; otherwise, a fixed-effect model was adopted. Publication bias was quantified using funnel plots and by computing the probability values obtained by the Egger (Egger et al., 1997) and Begg tests (Begg and Mazumdar, 1994).

## 3 Results

### 3.1 Study selection process

**Fig. 1:**
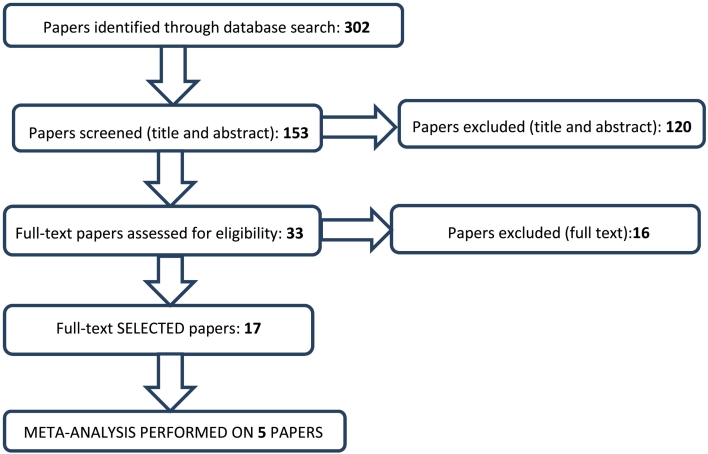
Flow chart representing the algorithm applied to select articles from the databases.

A search of the databases identified 302 papers (Fig. 1). After removing the duplicates, 153 papers were retained. Of these 153, 120 were excluded because they did not meet the inclusion criteria set, whereas the remaining 33 papers were retained for further analysis. Sixteen papers were excluded during the second screening stage which took the full text into account: 14 were excluded due to an absence of primary data in review papers or a lack of data (i.e. mathematical models), and two others due to unavailability of the full text (Supplementary Table S1). No papers were excluded for qualitative reasons. In the end, data were extracted from 17 papers, including data on baiting protocol and monitoring *E. multilocularis* prevalence (start-end prevalence) (Supplementary Table S2).

### 3.2 The initial trial

Among the 17 papers selected, the study by Rausch et al. (1990) in Alaska was the only paper to focus on dogs by administering praziquantel tablets and monitoring *E. multilocularis* in its intermediate hosts, rodents. This ten-year study, which started in 1980, was carried out in the isolated village of Savoonga (Saint Lawrence Island), where it succeeded in reducing the parasite's prevalence in rodents from 30% to 5%. Following this initial study in Alaska, the others were conducted in Europe (Germany, Switzerland, France, and the Slovak Republic) and in Japan, between 1988 and 2009, and were based on baiting foxes, monitoring *E. multilocularis* in foxes (feces and/or intestines), and occasionally also monitoring the parasite's prevalence in small rodents.

### 3.3 The fox baiting approach

With the exception of the study focused on dogs (Rausch et al., 1990), the other 16 included studies were related to fox baiting and carried out in a single area, several neighboring areas or even in different regions, constituting a total of 22 sub-studies. These sub-studies were performed both in urban and rural areas, generally corresponding to big differences in the size of the treated areas, respectively ranging between 1 and 213 km^2^, and between 156 and 4018 km^2^, apart from one study that involved baiting in a rural area of 6 km^2^ (Schelling and Frank, 1990). In the studies by Hegglin and Deplazes (2008) and Hegglin et al. (2003), several small plots (9 × 1 km^2^/1 × 6 km^2^ and 12 × 1 km^2^/2 × 6 km^2^ respectively) were used for baiting in the urban landscape of Zürich city and its surrounding areas. These studies evidenced a decrease in parasite prevalence and even total eradication in certain small areas. In contrast, two trials were carried out in Germany involving two large baiting areas: 566 km^2^ for Schelling et al. (1997) and 4450 km^2^ for Tackmann et al. (2001). Deworming was reported to have been more effective in central areas, where parasite prevalence in foxes decreased from 32% to 0% (Schelling et al., 1997) and from 16% to 6.2% (Tackmann et al., 2001). In peripheral areas, the prevalence decreased from 32% to 10% (Schelling et al., 1997) and from 1.7% to 0.9% (Tackmann et al., 2001). The authors attributed the lower effect of deworming to a higher number of infected foxes in these peripheral areas, no doubt having come from non-treated areas further away.

Two types of control area were used in order to quantify the effect of baiting on *E. multilocularis* prevalence. In five out of the 16 fox-baiting studies, the control and baiting areas were the same (Schelling and Frank, 1990; Schelling et al., 1997; Tackmann et al., 2001; Kamiya, 2007; Inoue et al., 2007). The prevalence of *E. multilocularis* was determined before treatment began in order to obtain an initial value with which to compare subsequent measurements. In this case, seasonal and annual variations in *E. multilocularis* prevalence may have distorted evaluations of the extent to which baiting was effective. In contrast, in the eleven other fox-baiting studies, the control area was different from the baiting area, although the same monitoring protocol for *E. multilocularis* prevalence and a similar plot size were adopted in both areas. Nevertheless, there may have been some differences between control and baiting areas in terms of landscape, urbanization or endemic level, particularly for large plots.

The method used to distribute the baits depended on the size of the area to treat: on foot when a few square kilometers were designated, and by car when the area was larger. In Germany, planes were used to distribute baits when the surface of the area to treat exceeded 200 km^2^. The only treatment used to target foxes involved distributing baits with praziquantel in the environment. This cesticidal drug had been reported as 100% effective against *E. multilocularis* when administered at 5 mg/kg of body weight in dogs (Andersen et al., 1981). A dose of 50 mg was used in foxes, since the average weight of these animals is estimated at between 6 and 10 kg. In the Japanese study, only 25 mg per bait (half of a Droncit® tablet) was used, and yet this quantity provided successful results (Tsukada et al., 2002). In nine studies, the bait density was 15–20 baits/km^2^ but higher densities (40 and 50 baits/km^2^) were used in five studies. A different approach was tested in Japan by distributing baits around identified fox dens (Takahashi et al., 2013; Tsukada et al., 2002).

Baiting lasted no more than a year in five of the 22 areas (sub-studies), indicating that parasite prevalence may have dropped sharply during this time. For the 12 trials lasting two years or more (up to five years and eight months), the prevalence fluctuations observed in foxes over these longer periods may be interpreted as resulting from seasonal variation of *E. multilocularis* prevalence.

The treatment frequency ranged from monthly to every six months. In four studies, the baiting frequency was varied during the course of treatment in order to observe its potential effect on *E. multilocularis* prevalence (Hegglin and Deplazes, 2008; König et al., 2008; Romig et al., 2007; Tackmann et al., 2001). In Germany, Romig et al. (2007) reported that prevalence in foxes decreased from 64% to 20% after baiting every six weeks over the course of a 17-month-long intervention. The prevalence remained stable throughout the following ten months with baits set every three months, but increased to 36% after 17 months when baits were set every six months. Once baiting stopped, *E. multilocularis* prevalence returned to its initial value within 16 months. A decrease in prevalence from 52% to 1% was achieved over three years by König et al. (2008), with baits initially distributed every four weeks but reduced to every six weeks once the infestation had significantly decreased. A reduction in parasite prevalence was also reported by Tackmann et al. (2001) in Germany, in both high (40% to 10% prevalence) and low (<10%) endemic areas, even after a reduction in baiting frequency from every six weeks during the first year to every 12 weeks during the last two years of the campaign. In urban areas of Zürich (Switzerland), Hegglin and Deplazes (2008) designed an experimental field study during which, over two successive phases of 1.5 and 2 years, baits were set at different frequencies (no baiting, monthly, every three months) in nine 1 km^2^ plots. *E. multilocularis* prevalence decreased to a low level after a monthly baiting period, while the three-monthly baiting period had a less notable effect.

### 3.4 Monitoring *E. multilocularis* prevalence

The effect of baiting was estimated by monitoring the prevalence of *E. multilocularis* in foxes. *E. multilocularis* worms were identified using the Sedimentation and Counting Technique (SCT) or the Intestinal Scraping Technique (IST) in Germany (König et al., 2008; Romig et al., 2007; Schelling and Frank, 1990; Schelling et al., 1997; Tackmann et al., 2001). The other European studies used the copro-ELISA test developed by Deplazes et al. (1999) or its commercial form, as developed by Antolová et al. (2006). In Japan, either necropsy or immunological techniques were adopted. In addition to monitoring prevalence in foxes, three trials (Hegglin et al., 2003; Schwarzenbach et al., 2004; Tsukada et al., 2002) macroscopically observed the presence of metacestodes in rodent livers and then confirmed these findings using PCR. The decreased prevalence observed in foxes was also observed in rodents, but with delayed action.

A decrease in *E. multilocularis* prevalence was reported in 17 of the 22 areas treated when comparing parasite prevalence in foxes at the start and end of the baiting period. In these successful trials, after at least one year of baiting and regardless of the treatment frequency, the prevalence in foxes generally dropped below 10%, even if the initial prevalence value was even higher than 30%. The prevalence was above 10% post treatment in only three Japanese studies carried out in extremely highly endemic areas of Hokkaido Island, where 49%, 57% and 60% of foxes were initially found to be infected and where post-treatment prevalences of 16%, 11% and 30% were respectively reached (Inoue et al., 2007; Takahashi et al., 2013; Tsukada et al., 2002). The baiting protocol's lack of effect in four areas could have been biased by variations in baiting frequency during distribution. For example, in the trial performed by Romig et al. (2007), a strong positive effect was reported after only 18 months but decreasing the baiting frequency to twice a year led to an increase in *E. multilocularis* prevalence. No significant effect was observed in two areas of the urban trial in Zürich (Hegglin and Deplazes, 2008). Such results were explained by the absence of baiting in the first of the two periods or by the decrease in baiting frequency from monthly to every three months. In the city of Pressov (Slovak Republic), a stable prevalence of 50% was reported in one of the two treated areas. Any positive effect of the treatment may have been masked by a simultaneous increase in parasite prevalence, which rose from 33% to 49% in the control area (Antolová et al., 2006). In the French city of Pontarlier, the absence of any significant decrease in parasite prevalence was attributed to the high risk of recontamination posed by infected foxes from outside the treated areas (Comte et al., 2013).

### 3.5 Meta-analysis results

**Fig. 2:**
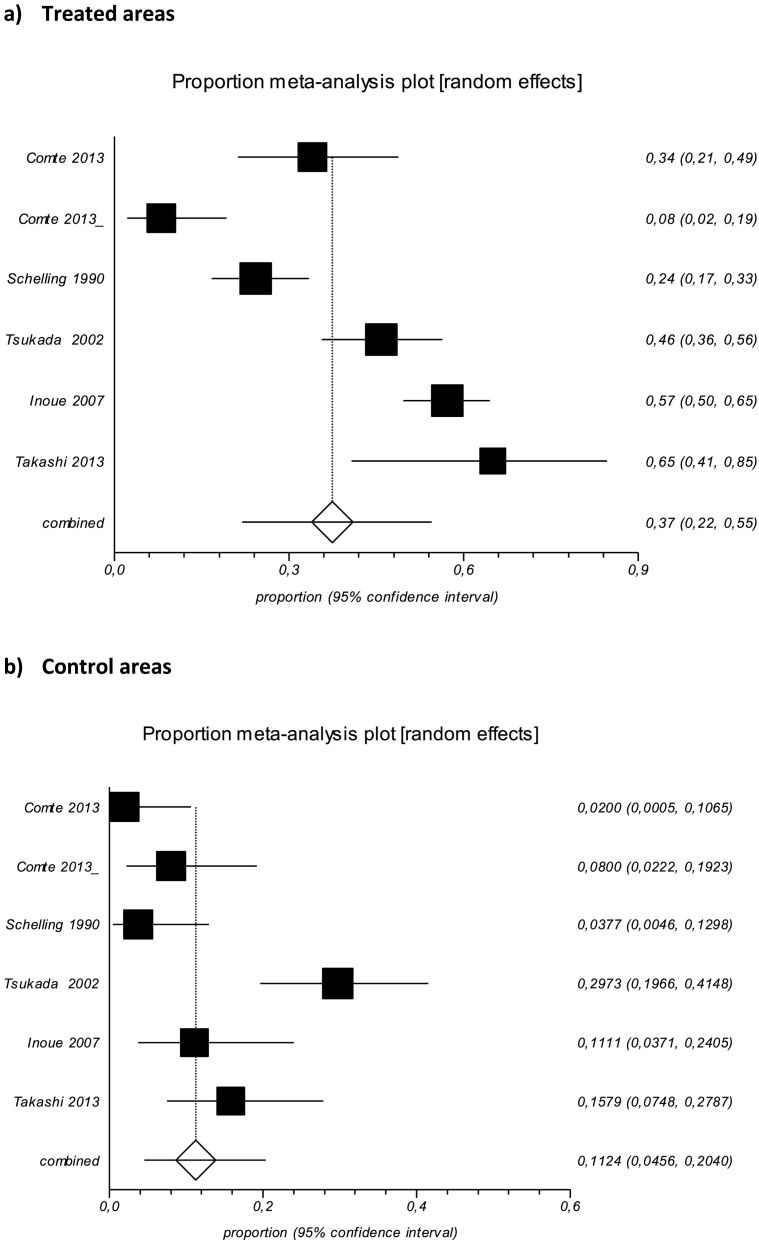
Forest plot for treated (a) and control (b) areas generated by software Stats Direct 2.8.0. **Treated areas**: Cochran Q p < 0.0001; I^2^ = 82%, Pooled proportion - random effect - = 0.112433 (95% CI = 0.04561 to 0.204014). **Control areas**: Cochran Q *p* < 0.0001; I^2^ = 93%, Pooled proportion - random effect - = 0.374968 (95% CI = 0.218974 to 0.545622).

A meta-analysis was performed based on the pooled risk difference, using data from five studies (Comte et al., 2013; Inoue et al., 2007; Schelling and Frank, 1990; Takahashi et al., 2013; Tsukada et al., 2002) and corresponding to six areas for which raw start-end prevalence data were available (Table 1, Table 2). The other eleven studies concerning fox baiting lacked raw data on the start prevalence. The pooled prevalence was estimated at 0.11% (95% CI = 0.05 to 0.20; *p* < 0.0001, *I*^*2*^ = 82%) in baiting areas (Table 1 and Fig. 2a) and 0.37% (95% CI = 0.22 to 0.55; *p* < 0.0001, *I*^*2*^ = 92.5%) in control areas (Table 2 and Fig. 2b). Where there was no overlap between confidence intervals, a significant difference was obtained, indicating that the baiting treatment was effective.

**Table 1:** Raw data from individual studies in treated areas.

First author's name	Reference	Year of publication	Country (city)	Chemical compound	Concentration in the bait	Frequency of treatment/year	Surface area in km^2^	Sample size at start	Positive samples at start	Sample size at end	Positive samples at end	Quality assessment (Cochrane)					
												A	B	C	D	E	F
Comte	Prev Vet Med; 111(1–2): 147–55.	2013	France (Annemasse)	Praziquantel	50 mg	5.25	33	50	NA	50	1	High	Low	Low	Low	Low	Low
Comte	Prev Vet Med; 111(1–2): 147–55.	2013	France (Pontarlier)	Praziquantel	50 mg	5.25	33	50	NA	50	4	High	Low	Low	Low	Low	Low
Schelling	Mitt Oesterr Ges Tropenmed Parasitol; 12: 185–191.	1990	Germany	Praziquantel	50 mg	3.6	6	53	2	–	–	High	Low	High	Low	Low	Low
Tsukada	Parasitology; 125(2): 119–29.	2002	Japan	Praziquantel	25 mg	12	90	156	93	74	22	Low	Low	Low	Low	Low	Low
Inoue	Vet Parasitol; 150(1–2): 88–96.	2007	Japan	Praziquantel	50 mg	12	110	56	32	45	5	High	Low	Low	Low	Low	Low
Takashi	Vet Parasitol; 198(1–2): 122–6.	2013	Japan	Praziquantel	50 mg	4.3	135	312	154	57	9	High	Low	Low	Low	Low	Low

A. sequence generation, B. allocation concealment, C. blinding, D. incomplete outcome data, E. selective outcome reporting, F. other sources of bias.

**Table 2:** Raw data from individual studies in control areas.

First author's name	Reference	Year of publication	Country	Surface area in km^2^	Sample size at start	Positive samples at start	Sample size at end	Positive samples at end	Quality assessment (Cochrane)					
									A	B	C	D	E	F
Comte	Prev Vet Med; 111(1–2): 147–55.	2013	France	160	50	NA	50	17	High	Low	Low	Low	Low	Low
Comte	Prev Vet Med; 111(1–2): 147–55.	2013	France	160	50	NA	50	4	High	Low	Low	Low	Low	Low
Schelling	Mitt Oesterr Ges Tropenmed Parasitol; 12: 185–191.	1990	Germany	6	111	27	–	–	High	Low	High	Low	Low	Low
Tsukada	Parasitology; 125(2): 119–29.	2002	Japan	110	129	54	96	44	Low	Low	Low	Low	Low	Low
Inoue	Vet Parasitol; 150(1–2): 88–96.	2007	Japan	110	180	103	–	–	High	Low	Low	Low	Low	Low
Takahashi	Vet Parasitol; 198(1–2): 122–6.	2013	Japan	277	95	67	20	13	High	Low	Low	Low	Low	Low

A. sequence generation, B. allocation concealment, C. blinding, D. incomplete outcome data, E. selective outcome reporting, F. other sources of bias.

**Fig. 3:**
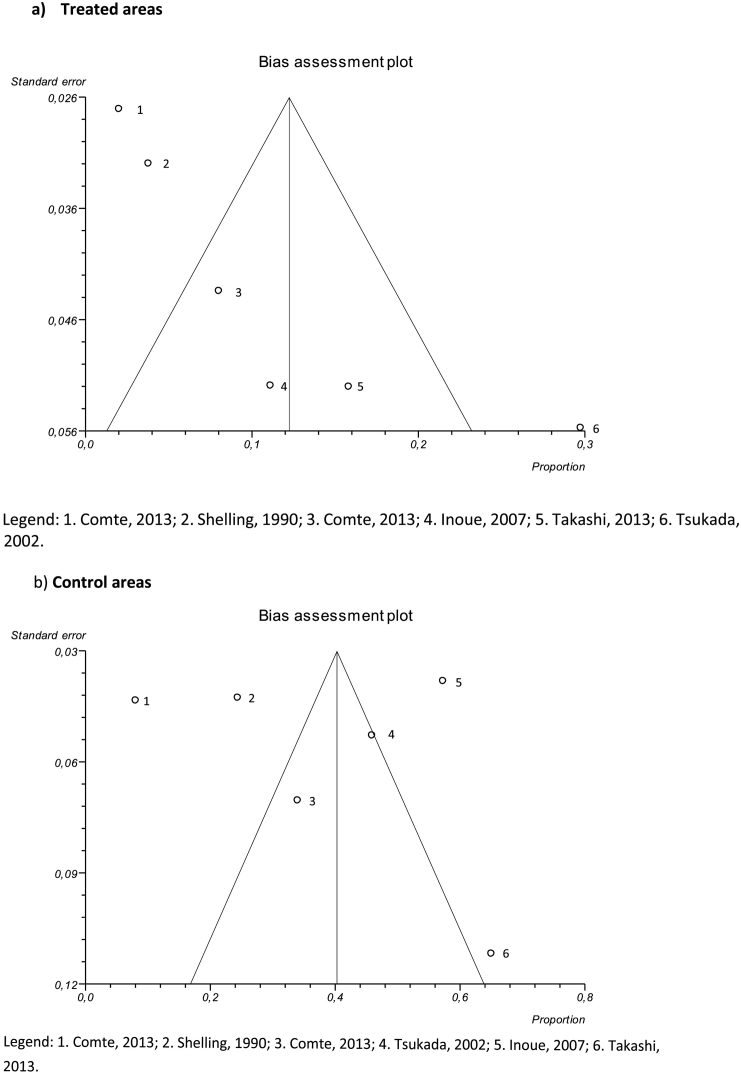
Funnel Plot for treated (a) and control (b) areas generated by software Stats Direct 2.8.0.

A funnel plot was used to evaluate publication bias. The plot showing studies performed in treated areas (Fig. 3a) showed a left symmetry. The funnel plot representing studies in control areas (Fig. 3b) displayed no asymmetry, suggesting that publication bias was unlikely.

In terms of quality assessment, all the studies included in this SR presented a high risk of bias in sequence generation while showing a low risk of bias in allocation concealment, incomplete outcome data, selective outcome reporting, and other sources of bias (Table 1).

## 4 Discussion

The use of fox baiting as a control program has generally been successful in decreasing *E. multilocularis* prevalence in foxes in both rural and urban areas, demonstrating the possibility of controlling the parasite on small to large scales. This overall positive effect of baiting was confirmed by the meta-analysis results, which showed a statistically significant decrease in parasite prevalence, signaled by the absence of overlapping confidence intervals for pooled risk differences between control and treated areas. The parasite was not eradicated in any of the programs, even though that was the initial purpose. Several factors may explain the parasite's persistence even at a very low prevalence level: (i) infection or re-infection of foxes soon after consuming the bait; (ii) deworming only targeting the parasite's adult stage in definitive hosts and not the metacestode stage in intermediate hosts or eggs in the environment; (iii) immigration of foxes from non-treated areas reducing the effect of baiting, notably along border areas, and re-introducing the parasite even in areas where it had theoretically been eradicated. Rather than a baiting strategy focusing on eliminating the parasite across large areas, Hegglin et al. (2003) recommended constant actions to lower the concentration of *E. multilocularis* eggs in defined risk areas, as this might be a more realistic and cost-efficient strategy. In this context, controlling *E. multilocularis* in urban areas might be less influenced by the border effect (Hegglin and Deplazes, 2008) since urban foxes tend to have small home ranges and low dispersal distances, as observed in the city of Zürich (Hegglin et al., 2003; Wandeler et al., 2003).

Initially, the bait density used for the trials was 20 baits/km^2^, as in rabies campaigns. Higher bait densities were later required due to fox populations having increased after rabies had been eradicated (König et al., 2008) or due to the higher fox densities observed in urban areas, exceeding 10 adult foxes/km^2^ (Hegglin et al., 2003). Consumption of the baits by non-targeted animal species (e.g. domestic dogs, wild boar, hedgehogs and stone martens) should also be taken into account when considering bait density.

A monthly baiting frequency has been proven efficacious in decreasing *E. multilocularis* prevalence in foxes, even in highly endemic areas and in a short period of time. This frequency enabled foxes to be dewormed during the prepatent period, without eggs being released (Kapel et al., 2006). After an initial monthly baiting period and once the prevalence had significantly decreased, a baiting frequency of every six weeks was successfully adopted by König et al. (2008). A greater decrease in frequency to baiting every three months proved to be of no use in maintaining low levels of environmental egg contamination on a small scale (Hegglin and Deplazes, 2008) but was successful on a larger scale (Romig et al., 2007). These findings support the results obtained by mathematical models of *E. multilocularis* control, which had indicated that baiting intervals of 4 to 6 weeks would be the most efficacious (Hansen et al., 2003) because, with monthly treatment, it can take several years for the parasite to recolonize a small-scale baiting area (Hegglin and Deplazes, 2008). Nevertheless, when the parasite was not completely eradicated, it had a strong capacity to rapidly return to its initial prevalence, as observed in Germany where there was an increase from 15% to 64% in 16 months without baiting (Romig et al., 2007). These findings highlight the necessity of permanent baiting to maintain low prevalence and potentially eradicate the parasite over a longer period.

Monitoring *E. multilocularis* prevalence is essential to evaluating the efficacy of baiting trials. Both necropsy and copro-antigen detection approaches have been shown to be suitable for monitoring *E. multilocularis* prevalence in foxes in this context. Intestinal diagnostic techniques such as SCT are relatively cheap when only considering the cost of consumables. The techniques are also highly specific and the SSCT (Segmental Sedimentation and Counting Technique), a variant of the SCT gold standard, is far less time-consuming (Umhang et al., 2011). Nevertheless, it requires sampling foxes resident in treated areas, which may affect the final result due to the possible intrusion of neighboring foxes from non-treated areas (Tsukada et al., 2002). On the other hand, copro-ELISA analyses are less specific and commercial kits are no longer available. Nevertheless, as feces are a non-invasive sampling method and a proxy for environmental contamination with *E. multilocularis* eggs, the recently-developed copro-qPCR assays may provide a new monitoring approach (Knapp et al., 2014; Knapp et al., 2016).

Over the two decades following the first baiting trial in 1988, many baiting control programs targeting *E. multilocularis* were carried out, but since 2009 no further trials have been implemented. Although there was a decrease in *E. multilocularis* prevalence among foxes in almost all of these experimental studies, the cost of these trials has never been properly evaluated. The cost of baiting five times per year has been estimated at €114 for a 1 km^2^ plot, when carried out over 32 months in a medium-sized city in France, excluding the cost of copro-ELISA monitoring (Comte et al., 2013). The cost may be reduced by 40% through involving local technical staff in bait distribution. It is furthermore essential to put control program costs into perspective: in Switzerland, treating one human case of AE is estimated to cost €108,762 (CI: €48,302–€178,568) (Torgerson et al., 2008). Hegglin and Deplazes (2013) evaluated the cumulated costs and benefits of an anthelmintic fox treatment in different scenarios ranging from small to large areas and corresponding to decreasing human population density. After an initial phase of monthly baiting to decrease *E. multilocularis* prevalence, a second phase could be scheduled. Although baiting is constant throughout this phase, the baiting frequency is reduced. Cost-effectiveness was considered to be highest in small urban endemic areas (100 km^2^, 4000 people/km^2^) with a total cost per inhabitant of €1.27 after long-term baiting lasting 20 years and reached a negative cost of €-0.98 after 40 years. Nevertheless, it is essential to further optimize species-specific baiting systems and to design new mathematical models (Hegglin and Deplazes, 2013).

## 5 Conclusion

Despite the high quality of the studies performed, this systematic review highlights an absence of raw data from baiting control programs (numbers of samples tested, numbers of *E. multilocularis*-positive animals) in many studies. The lack of these raw data resulted in these papers being excluded from the meta-analysis.

Baiting programs have proved to be efficacious in significantly decreasing the prevalence of *E. multilocularis* in red foxes, but they must form part of an integrated prevention campaign against AE, including informing the public and regularly deworming dogs. Since baiting programs require a time commitment of several decades, an accurate and specific cost-benefit analysis would be required in order for decision-makers to agree to invest in such long-term public health measures. This analysis should take into account the costs involved in treating human cases before considering the cost of implementing such programs.

The following are the supplementary data related to this article.

Supplementary Table S1 Sixteen excluded papers from the systematic review by full text check. Supplementary Table S1

Supplementary Table S2 Seventeen included papers in the systematic review. In bold papers entered in meta-analysis. Supplementary Table S2
