# Supplementary material for: A systematic review and meta-analysis on anthelmintic control programs for Echinococcus multilocularis in wild and domestic carnivores
Source: Food Waterborne Parasitol. 2019 Mar 14;15:e00042. doi: 10.1016/j.fawpar.2019.e00042 (PMC7034083; doi:10.1016/j.fawpar.2019.e00042)
Supplement: Supplementary Table S1 — Sixteen excluded papers from the systematic review by full text check. [file mmc1.docx]

**Supplementary Table S1-** Sixteen excluded papers from the systematic review by full text check.

| **Authors** | **Reference** | **Title** | **Reason for exclusion** |
| --- | --- | --- | --- |
| Bessonov, A. S. | NATO Science Series: Life and Behavioural Sciences, Vol. 341. Proceedings of the NATO Advanced Research Workshop on cestode zoonoses: echinococcosis and cysticercosis: an emergent and global problem, Poznan, Poland, 10-13 September 2000 (2002), pp. 91-98. | Echinococcoses of animals and humans in the Russian Federation. | Review |
| Duscher, G.; Steineck, T.; Guenter, P.; Prosl, H.; Joachim, A. | Wiener Tieraerztliche Monatsschrift (2005), Vol. 92 (1): 16-20 | *Echinococcus multilocularis* in foxes in Vienna and surrounding territories. | No Data on baiting strategy |
| Eckert, J.; Gemmell, M. A.; Soulsby, E. J. L. | FAO/UNEP/WHO guidelines for surveillance, prevention and control of echinococcosis/hydatidosis. (1981), ix + 147 p., Document VPH/81.28; 27 contributors | FAO/UNEP/WHO guidelines for surveillance, prevention and control of echinococcosis/hydatidosis. | Review |
| Economides, P.; Christofi, G. | NATO Science Series: Life and Behavioural Sciences, Vol. 341. Proceedings of the NATO Advanced Research Workshop on cestode zoonoses: echinococcosis and cysticercosis: an emergent and global problem, Poznan, Poland, 10-13 September 2000 (2002), pp. 367-379. | Experience gained and evaluation of the echinococcosis/hydatidosis eradication programmes in Cyprus 1971 - 1999. | Data on E. granulosus |
| Hansen F.; Tackmann K.; Jeltsch F.; Thulke H.-H. | Berliner und Munchener tierarztliche Wochenschrift, (200) Vol. 116 (7-8): 299-305. | [Baiting intervals and duration of control of the small fox tapeworm: a simulation study]. | Mathematical model |
| Hegglin, D.; Bontadina, F.; Gloor, S.; Romer, J.; Mueller, U.; Breitenmoser, U.; Deplazes, P. | Journal of Wildlife Management, (2004) Vol. 68 (4): 1010-1017. | Baiting red foxes in an urban area: A camera trap study. | No Data on Baiting strategy |
| Hegglin D.; Deplazes P. | International journal for parasitology, (2013) Vol. 43 (5): 327-37. | Control of *Echinococcus multilocularis*: strategies, feasibility and cost-benefit analyses. | Review |
| Hinz E. | Zentralblatt fur Bakteriologie, Parasitenkunde, Infektionskrankheiten und Hygiene. Erste Abteilung Originale. Reihe A: Medizinische Mikrobiologie und Parasitologie, (1978 Jun) Vol. 240 (4): 542-8. | [Fenbendazole therapy of experimental larval echinococcosis. I. The effect of fenbendazole on worm burden and protoscolex development of *Echinococcus multilocularis*]. | Animal experimentation |
| Ito A; Romig T; Takahashi K | Parasitology, (2003) Vol. 127 Suppl: S159-72. | Perspective on control options for *Echinococcus multilocularis* with particular reference to Japan. | Review |
| Kamiya M.; Lagapa J. T. G.; Nonaka N.; Ganzorig S.; Oku Y.; Kamiya H. | Revue scientifique et technique (International Office of Epizootics), (2006) Vol. 25 (3): 1055-65. | Current control strategies targeting sources of echinococcosis in Japan. | Review |
| König, A.; Janko, C.; Barla-Szabo, B.; Fahrenhold, D.; Heibl, C.; Perret, E.; Wermuth, S. | Wildlife Research, (2012) Vol. 39 (6): 488-495. | Habitat model for baiting foxes in suburban areas to counteract *Echinococcus multilocularis*. | Mathematical model |
| Roberts M. G.; Aubert M. F | Veterinary parasitology, (1995) Vol. 56 (1-3): 67-74. | A model for the control of *Echinococcus multilocularis* in France. | Mathematical model |
| Schantz, P. M. | Report of the the PAHO/WHO Working Group on perspectives and possibilities of control and eradication of hydatidosis (2002), pp. 62-74. | Echinococcosis world distribution and prospects for control. | Mathematical model |
| Takumi K.; Van der Giessen J. | Parasitology, (2005) Vol. 131 (Pt 1): 133-40. | Transmission dynamics of *Echinococcus multilocularis*; its reproduction number, persistence in an area of low rodent prevalence, and effectiveness of control. | Mathematical model |
| Gemmell, M. A. | Compendium on cystic echinococcosis: with special reference to the Xinjiang Uygur Autonomous Region, The People's Republic of China. (1993): 57-73 | Quantifying the transmission dynamics of the family Taeniidae with particular reference to *Echinococcus* spp. | Missing paper |
| Mix, H. | (2001), 203 p [Livre] | [Epidemiological study of the effectiveness of a scheme to control *Echinococcus multilocularis* among free-living foxes, and the occurrence of other intestinal helminths of foxes in northwestern Brandenburg, Germany]. | Missing paper |
