# Supplementary material for: A systematic review and meta-analysis on anthelmintic control programs for Echinococcus multilocularis in wild and domestic carnivores
Source: Food Waterborne Parasitol. 2019 Mar 14;15:e00042. doi: 10.1016/j.fawpar.2019.e00042 (PMC7034083; doi:10.1016/j.fawpar.2019.e00042)
Supplement: Supplementary Table S2 — Seventeen included papers in the systematic review. In bold papers entered in meta-analysis. [file mmc2.docx]

**Supplementary Table S2 –** Seventeen included papers in the systematic review. In **bold** papers entered in meta-analysis.

| **Authors** | **Reference** | **Title** | **Year** |
| --- | --- | --- | --- |
| Antolova, D.; Miterpakova, M.; Reiterova, K.; Dubinsky, P. | Helminthologia (Bratislava), Vol. 43(4): 226-231. | Influence of anthelmintic baits on the occurrence of causative agents of helminthozoonoses in red foxes (*Vulpes vulpes*). | 2006 |
| **Comte S.; Raton V.; Raoul F.; Hegglin D.; Giraudoux P.; Deplazes P.; Favier S.; Gottschek D.; Umhang G.; Boue F.; Combes B.** | **Preventive veterinary medicine, Vol. 111 (1-2): 147-55.** | **Fox baiting against *Echinococcus multilocularis*: contrasted achievements among two medium size cities.** | **2013** |
| Hegglin D.; Deplazes P. | Emerging infectious diseases, Vol. 14 (10): 1626-8. | Control strategy for *Echinococcus multilocularis*. | 2008 |
| Hegglin D.; Ward P. I.; Deplazes P. | Emerging infectious diseases, Vol. 9 (10): 1266-72. | Anthelmintic baiting of foxes against urban contamination with *Echinococcus multilocularis*. | 2003 |
| König, A.; Romig, T.; Janko, C.; Hildenbrand, R.; Holzhofer, E.; Kotulski, Y.; Ludt, C.; Merli, M.; Eggenhofer, S.; Thoma, D.; Vilsmeier, J.; Zannantonio, D. | European Journal of Wildlife Research, Vol. 54 (3): 439-447. | Integrated-baiting concept against *Echinococcus multilocularis* in foxes is successful in southern Bavaria, Germany. | 2008 |
| Tackmann K; Loschner U; Mix H; Staubach C; Thulke H H; Ziller M; Conraths F J | Epidemiology and infection, Vol. 127 (3): 577-87. | A field study to control *Echinococcus multilocularis*-infections of the red fox (*Vulpes vulpes*) in an endemic focus. | 2001 |
| **Inoue, T.; Nonaka, N.; Kanai, Y.; Iwaki, T.; Oku, Y.; Kamiya, M.** | **Veterinary Parasitology, Vol. 150 (1-2): 88-96.** | **The use of tetracycline in anthelmintic baits to assess baiting rate and drug efficacy against *Echinococcus multilocularis* in foxes.** | **2007** |
| Janko C.; Konig A. | Journal of wildlife diseases, Vol. 47 (2): 373-80. | Disappearance rate of praziquantel-containing bait around villages and small towns in southern Bavaria, Germany. | 2011 |
| Kamiya M. | Journal of veterinary science, Vol. 8 (4): 313-21. | Collaborative control initiatives targeting zoonotic agents of alveolar echinococcosis in the northern hemisphere. | 2007 |
| Nonaka N.; Kamiya M.; Oku Y. | Parasitology international, Vol. 55 Suppl: S263-6. | Towards the control of *Echinococcus multilocularis* in the definitive host in Japan | **2006** |
| Rausch, R. L.; Wilson, J. F.; Schantz, P. M. | Annals of Tropical Medicine and Parasitology, Vol. 84 (3): 239-250. | A programme to reduce the risk of infection by *Echinococcus multilocularis*: the use of praziquantel to control the cestode in a village in the hyperendemic region of Alaska | **1990** |
| Romig, T.; Bilger, B.; Dinkel, A.; Merli, M.; Thoma, D.; Will, R.; Mackenstedt, U.; Lucius, R. | Helminthologia (Bratislava), Vol. 44 (3): 137-144. | Impact of praziquantel baiting on intestinal helminths of foxes in southwestern Germany. | 2007 |
| **Schelling, U.; Frank, W.** | **Mitteilungen der Oesterreichischen Gesellschaft fuer Tropenmedizin und Parasitologie, Vol. 12: 185-191.** | **Attempt to eliminate *Echinococcus multilocularis* in the definitive host by anthelmintic prepared baits.** | **1990** |
| Schelling U.; Frank W.; Will R.; Romig T.; Lucius R. | Annals of tropical medicine and parasitology, Vol. 91 (2): 179-86. | Chemotherapy with praziquantel has the potential to reduce the prevalence of *Echinococcus multilocularis* in wild foxes (*Vulpes vulpes*). | 1997 |
| Schwarzenbach G. A.; Hegglin D.; Stieger C.; Deplazes P.; Ward P. I. | Parasitology, Vol. 129 (Pt 1): 93-9. | An experimental field approach to parasitism and immune defence in voles. | 2004 |
| **Takahashi, K., Uraguchi, K., Hatakeyama, H., Giraudoux, P., Romig, T.** | **Veterinary Parasitology**. **198 (1-2): 122-126.** | **Efficacy of anthelmintic baiting of foxes against *Echinococcus multilocularis* in northern Japan** | **2013** |
| **Tsukada H; Hamazaki K; Ganzorig S; Iwaki T; Konno K; Lagapa J T; Matsuo K; Ono A; Shimizu M; Sakai H; Morishima Y; Nonaka N; Oku Y; Kamiya M** | **Parasitology, Vol. 125 (Pt 2): 119-29.** | **Potential remedy against *Echinococcus multilocularis* in wild red foxes using baits with anthelmintic distributed around fox breeding dens in Hokkaido, Japan.** | **2002** |
